# Cancer services patient experience in England: quantitative and qualitative analyses of the National Cancer Patient Experience Survey

**DOI:** 10.1136/spcare-2022-003543

**Published:** 2022-06-29

**Authors:** Gavin Brookes, Paul Baker

**Affiliations:** Linguistics and English Language, Lancaster University, Lancaster, UK

**Keywords:** Cancer, Hospital care, Service evaluation, Communication

## Abstract

**Objectives:**

To examine patients’ responses to the English National Cancer Patient Experience Survey to understand what proportions of patients give positive and negative feedback, and to identify themes in responses which drive evaluations.

**Methods:**

Data comprise 214 340 survey responses (quantitative ratings and free-text comments) dated 2015–2018. The proportions of patients giving each quantitative rating (0–10) are compared and free-text comments are analysed using computer-assisted linguistic methods in order to ascertain frequent thematic drivers of positive and negative feedback.

**Results:**

Patients were most likely to give a most positive score of 10 (38.25%), while the overwhelming majority (87.12%) gave a score between 8 and 10. Analysis of 1000 positive comments found that most respondents (54%) praised staff’s interpersonal skills. Other frequent themes of positive feedback included treatment standards, staff’s communication skills, speed of diagnosis and treatment, and staff members’ technical competence. The most prominent themes in the negative comments were communication skills, treatment standards and waiting times for appointments and test/scan results, and delays and cancellations to appointments and operations.

**Conclusion:**

Standards of treatment and staff’s communication skills are prominent themes of positive and negative feedback. Staff’s interpersonal skills are more likely to be praised than criticised, while negative feedback is more likely to focus on issues around time (ie, delays and long waits). Clarity and honesty in communication about the lengths and causes of waits and delays are likely to increase patient satisfaction.

**WHAT IS ALREADY KNOWN ON THIS TOPIC**

- General factors influencing patient satisfaction, including how treatment stage and cancer type influence satisfaction rates.

**WHAT THIS STUDY ADDS**

- This study provides new and up-to-date insight into satisfaction rates and themes driving evaluations.
- Feedback from patients tends to be very positive, with the highest score (10) being the most commonly given and with 87.12% of respondents giving a score of at least 8 out of 10.
- Treatment standards and communication drive both positive and negative feedback.
- Interpersonal skills tend to drive praise, while issues around time tend to drive criticism.

**HOW THIS STUDY MIGHT AFFECT RESEARCH, PRACTICE AND/OR POLICY**

- Improved communication regarding lengths and causes of delays is likely to improve patient satisfaction rates in cancer care.
- Researchers should be mindful that quantitative ratings given in feedback influence the themes raised in qualitative comments.

## Introduction

This study examines quantitative and qualitative patient feedback on National Health Service (NHS) cancer care services in England, focusing on the former in terms of overall experience and satisfaction rates and the latter in terms of frequent themes of positive and negative evaluations. Patient satisfaction can be defined as ‘an individual patient or family visitor’s subjective perspective on medical services received […] adopted as one of the indicators of care quality’ (p122).^1^

Patient feedback exercises are an established way for healthcare organisers to understand patients’ experiences of healthcare services, with such understanding increasingly viewed as critical to monitoring and improving the quality of those services. Finding ways to deliver ‘high-quality, person-centred care is central to [NHS] policy, and has been driven by rising demands, financial pressures, concerns about standards of care and a greater focus on the ‘consumer’s’ perspective’ (2, p1).^3^ Patient experience is now widely recognised, alongside clinical effectiveness and safety, as a critical element of high-quality healthcare, while patient satisfaction has been shown to be ‘positively associated with a range of health, resource use and safety outcomes’ (p1).^2^ Embedding the results of patient satisfaction and experience surveys into care delivery has been shown to lead to improved understanding of patients’ expectations,^4^ which in turn can result in improved health outcomes for patients.^5^

Cancer is a leading cause of death worldwide.^6^ In the UK, one in two people will be affected by cancer during their lifetime.^7^ For many patients with cancer, being diagnosed and treated ‘is a long and complicated process, involving multiple stages of investigation and treatment, and multiple encounters with a variety of health professionals and services’ (p1).^2^ According to the WHO, 34% of adults in need of palliative care require it due to cancer.^8^ Richard and colleagues^3^ underscore the particular need to evaluate patient satisfaction and experience with cancer care because of the diversity of patients, patients’ complex care needs and the increased survivorship of people diagnosed with cancer. For the NHS in England—the healthcare system under focus in the present study—patient experience features among four key metrics used to rate cancer care services commissioned by local systems.^9^

Relative to the volume of research on general patient feedback, studies of patient satisfaction with and experiences of cancer care services are few in number. Most existing studies have considered patients’ experiences at particular points along the care trajectory, for example during follow-up^10^ and in hospital,^11^ or among patients with particular types of cancer.^12 13^ Variables that have been found to influence patients’ satisfaction with cancer care include waiting times, availability and frequency of contact with healthcare providers, interpersonal aspects of care, patient-centred care, continuity of care and the physical environment in which care is provided.^3^ Corner *et al* 14 carried out a qualitative content analysis on free-text responses to a postal questionnaire on experiences of cancer treatment services in England. Approximately one-fifth (19%) of their respondents described experiences of excellent care during the treatment phase, while just 8% reported negative experiences. However, they also found that most respondents related negative experiences of care after primary cancer treatment. More recently, Cunningham and Wells^2^ examined free-text responses to the first Scottish Cancer Patient Experience Survey. Their inductive thematic analysis of 6961 free-text comments, provided by 4835 respondents, highlighted the importance to patients of feeling that their individual needs were met and feeling confident with the system. Their analysis also highlighted how processes and structures within the system of care could negatively impact patients’ experience, while particular issues were identified with care experiences leading up to the point of diagnosis.

This paper reports on the analysis of feedback provided by the respondents of the NHS England’s Cancer Patient Experience Survey between 2015 and 2018 (inclusive). The purpose of our analysis was to understand overall rates of patient experience based on qualitative feedback. In examining the themes that characterise positive feedback, we aim to understand what patients valued about their experiences of cancer care services. In examining the themes characterising negative feedback, we aim to highlight those themes which indicate areas for potential service improvement. This study aims to build on previous work in this area in the following ways. The patient feedback data examined in this paper constitute, to our knowledge, the largest and most recent collection of patient feedback on UK cancer care services to be studied. Given the ever-changing landscape of healthcare provision in England, this study therefore importantly responds to the need for regular and up-to-date research that assesses patient experiences in this context. Furthermore, where previous studies have, as noted, tended to focus on feedback from patients with particular types of cancer or at particular stages in their treatment, the data analysed in this study comprise responses from England’s Cancer Patient Experience Survey (discussed in the next section), and so represents feedback from patients experiencing different types of cancer who have received treatment for different durations.

## Methods

### Data

The data examined in this study consist of the feedback written by participants of England’s Cancer Patient Experience Survey. This survey is sent annually by NHS England to all patients who receive treatment for cancer. Responses were provided both online and using pen-and-paper forms, with the latter subsequently digitised to render it amenable to computational analysis. The feedback obtained is both quantitative and qualitative. The quantitative component asks respondents the following: ‘Overall, how would you rate your care?’, to which they can respond by providing a score between 0 and 10, where at one end of the spectrum a score of 0 indicates a very negative evaluation and at the other end of the scale a score of 10 indicates a very positive evaluation. Respondents then had the opportunity to describe their experiences and to explain why they gave the score they did by making use of three free-text boxes on the form. These free-text boxes are preceded by the following questions: ‘was there anything particularly good about your NHS cancer care?’, ‘was there anything that could have been improved?’ and ‘any other comments?’ Our analysis examines both the quantitative and qualitative components of the form described above.

This study is based on an analysis of 214 340 responses (each comprising a respondent’s comments to all of the questions comprising the survey) provided between 2015 and 2018 (the data made available to the researchers by NHS England’s Insight and Feedback team). The feedback represents patients’ experiences across all NHS hospitals based in England that provide adult cancer services and which took part in the survey. Table 1 gives a breakdown of the data and respondents’ characteristics.

**Table 1:** Breakdown of feedback

	%		%
**Age**		**Ethnicity**	
16–24	0.32	African	0.53
25–34	1.08	Arab	0.09
35–44	3.26	Bangladeshi	0.09
45–54	10.91	Caribbean	0.67
55–64	21.65	Chinese	0.22
65–74	36.08	English/Welsh/Scottish/Northern Irish	87.09
75–84	22.36	Gypsy or Irish Traveller	0.02
85+	4.34	Indian	0.98
		Irish	1.03
**Sex**		Pakistani	0.34
Female	54.38	White and Asian	0.20
Male	45.62	White and black African	0.08
Unspecified	<0.00	White and black Caribbean	0.23
		Any other Asian background	0.45
**Sexuality**		Any other black/African/Caribbean background	0.08
Bisexual	0.27	Any other ethnic group	0.24
Gay or lesbian	0.73	Any other mixed background	0.16
Heterosexual	90.86	Any other white background	2.07
Other	0.32	No answer	5.43
Prefer not to say	1.87		
No answer	5.95	**Length of treatment**	
		Less than 1 year	60.09
**English as first language**		1–5 years	27.80
No	3.50	More than 5 years	7.65
Yes	92.40	Don’t know/no answer	4.46
No answer	4.10		
		**Year**	
		2015	24.57
		2016	25.16
		2017	24.40
		2018	25.87

### Analytical approach

The first part of our analysis is quantitative and simply compares the number of patients who gave each of the quantitative ratings (0–10) in order to get an impression of patient satisfaction rates overall. The second part of our analysis aims to provide insight into the key themes of positive and negative feedback by focusing on frequent themes of each type of evaluation emerging from the qualitative feedback given in the free-text boxes. This part of our analysis uses techniques from corpus linguistics. This essentially refers to a collection of computer-aided methods which help human analysts to identify recurrent and statistically salient patterns of language use in large, digitised collections of text.^15^ Supported by specialist computer software, corpus linguistic methods allow human analysts to account for patterns in language use in large and more widely representative samples of text than would be possible through manual analysis alone, thereby arriving at more generalisable conclusions about the linguistic and thematic properties of the types of texts under study. Corpus linguistic methods are well suited to analysing the large volume of patient feedback data under focus in this study, which amounts to 14 403 694 words, and such methods have previously been used to examine recurrent themes, as indicated through the repeated use of particular words and strings of words, in similar patient feedback texts.^16 17^ We began by dividing the comments into three sets according to the section of the feedback form they were written in response to the following: (1) was there anything particularly good about your NHS care?, (2) was there anything that could have been improved? and (3) any other comments? Our qualitative analysis of the free-text comments focuses on the first two of these data sets, as these correspond broadly to positive and negative feedback, respectively. The positive subset comprises 190 168 comments (5 471 549 words) and the negative subset contains 139 178 comments (5 615 658 words).

We then took the comments responding to question 1, which focuses overwhelmingly on themes of positive evaluation, and using the CQPweb corpus analysis tool^18^ we obtained a list of the 10 most frequent evaluation words in these comments. To ensure that these words were used to perform evaluations, we manually checked random samples of 500 of their uses. We then carried out the same procedure on the negative comments provided in response to question 2, identifying the 10 most frequent negative evaluation words in these texts. These words are given in the Results section. We then used the top 10 positive and top 10 negative evaluation words as an entry point through which to qualitatively analyse the most frequent themes of positive and negative evaluations based on their respective sets of comments. Specifically, we analysed 100 uses of each of the words (a total of 1000 positive comments and 1000 negative comments) and coded the comments for the themes that drove patients’ positive and negative evaluations. We adopted an inductive approach, with the development of codes being driven by the comments themselves. Codes were checked by both authors to ensure consistency, and problematic cases were discussed until both authors reached an agreement on the code(s) eventually applied. Comments containing no explicit evaluation (ie, which provided ostensibly factual or neutral accounts) were excluded from the sample, as were vague comments from which no specific theme of evaluation could be ascertained (eg, “Everyone was very good and I was very pleased”). Comments were assigned as many codes as was necessary to reflect the themes in the comment.

## Results

### Analysis of quantitative feedback

**Figure 1:**
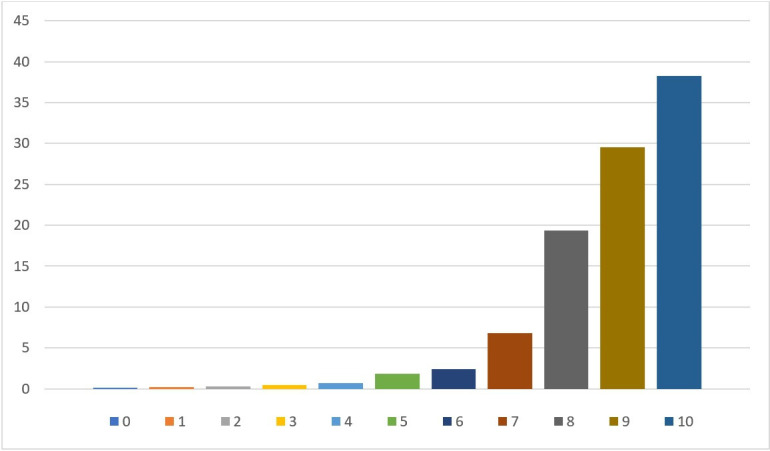
Percentage of respondents who gave each score between 0 and 10.

The first step in our analysis was to consider the proportions of patients who gave each of the quantitative ratings between 0 and 10. This information is presented in table 2 and figure 1. Note not all respondents provided a rating in their feedback, although the majority did (96.32%). These figures are therefore based on the feedback from respondents who did provide a rating as part of their feedback.

**Table 2:** Number of respondents who gave each rating

Rating	Respondents (n)	% of feedback
0	295	0.14
1	460	0.22
2	529	0.26
3	953	0.46
4	1436	0.70
5	3846	1.86
6	4998	2.42
7	14 129	6.84
8	39 877	19.32
9	60 958	29.53
10	78 965	38.25

As table 2 and figure 1 indicate, patients who provided free-text feedback were much more likely to give positive than negative experience and satisfaction scores, with the highest rating of 10 receiving the largest proportion of comments (38.25%), while 87.12% of patients gave a score of 8 or above. In fact, the number of respondents giving each score rises consistently from 0 all the way up to 10, with 0 being the least commonly given score (0.14%) and only 1.78% of respondents giving a score in the bottom half of the scale (ie, of 4 or below).

### Analysis of qualitative feedback

The quantitative ratings that patients gave is only part of the picture and respondents also had the option to explain the score they gave by leaving a qualitative feedback in the free-text boxes. As described in the Methods section, our qualitative analysis of the feedback is based on samples of comments posted to these free-text boxes (1000 positive, 1000 negative), obtained by searching for 100 randomly selected uses of each of the most frequent positive and negative evaluation words, respectively. These words are shown in table 3.

**Table 3:** Ten most frequent positive and negative evaluation words

Rank	Positive		Negative	
	Word	Frequency	Word	Frequency
1	excellent	40 021	poor	4081
2	good	39 093	problem	3813
3	brilliant	7634	wrong	2410
4	great	7592	bad	2315
5	fantastic	6999	issues	1170
6	wonderful	6739	issue	1106
7	amazing	6213	concerned	1093
8	best	6183	complaint	1037
9	outstanding	4088	worse	814
10	exceptional	3365	unnecessary	801

Beginning with the positive feedback, our analysis of 1000 comments, based on the words on the left side of table 3, uncovered a wide range of themes of positive evaluation. The five most frequent themes are shown in table 4, which also provides representative extracts to exemplify each theme. The themes in this table were all found in at least 10% of the comments analysed.

**Table 4:** Top 5 themes of positive feedback

Rank	Theme	Found in % of comments	Example extract
1	Interpersonal skills	54	“The care I received was excellent. They were kind and considerate.”
2	Treatment	29	“Then at the [anon] Hospital, the surgeon [anon] was wonderful and ensured he and his team gave me a first class service in all aspects - pre-operation, operation itself and also aftercare.”
3	Communication skills	20	“All information was explained if I didn’t understand, and all questions were answered quickly and succinctly.”
4	Speed of diagnosis and treatment	14	“I appreciate my consultant at [anon] Hospital and his/her staff for acting so quickly, they saved my life.”
5	Staff’s technical competence	10	“I cannot thank [anon] chemotherapy unit enough for the care that I have been given this year. All staff are amazing, so kind, caring and knowledgeable.”

As table 4 indicates, positive feedback is driven mostly by staff’s interpersonal skills, which were noted by patients as a reason for praise in over half (54%) of the comments in the sample. This strong focus on staff members’ interpersonal skills is reflected in some of the most frequent evaluative words in this set of comments, which we excluded from the analysis as they would have resulted in our focus being skewed towards comments focusing on interpersonal skills. Nevertheless, these terms indicate the types of attributes that led to staff members being evaluated positively for their interpersonal skills (frequencies in brackets): *caring* (24 800), *helpful* (16 008), *kind* (12 184) and *friendly* (10 699).

Linked to interpersonal skills, staff’s communication skills drove evaluations in one-fifth (20%) of positive comments. In these cases, respondents praised staff who communicated with patients clearly and frequently about their care and what that would entail. Comments praising communication also related to how well (and sensitively) diagnoses were communicated to patients, and this was frequently linked to staff having strong interpersonal skills.

Not all of the themes could be linked to social and communication skills, though, as almost one-third (29%) of the positive comments analysed focused on the standards of treatment, reflecting patient satisfaction with the outcomes of treatment or the manner in which treatment was carried out. Relatedly, 10% of respondents praised staff for their technical competence. Like communication and interpersonal skills, respondents frequently praised standards of treatment and staff’s interpersonal skills together, ostensibly perceiving these to be linked.

Finally, 14% of respondents praised the speed or efficiency of the treatment provided, including timely diagnosis, and then the short period between diagnosis and start of treatment.

Moving on to the negative feedback, we carried out the same procedure as above, this time examining 1000 negative comments obtained by randomly selecting 100 comments using each of the negative evaluation words in table 3. Table 5 displays the results of this analysis, focusing on the top 5 themes of negative feedback.

**Table 5:** Top 5 themes of negative feedback

Rank	Theme	Found in % of comments	Example
1	Communication skills	37	“Poor communication skills. No eye contact, looking at computer screens instead of patient.”
2	Appointment/operation waiting times	22	“Admin and arrival of treatment in day care was poor and haphazard, resulting in long waits when still feeling very poorly.”
3	Treatment	18	“I must stress that the negative aspect prior to starting my treatment at the hospitals in question, relative to the period leading up to my original diagnosis is directly due to poor treatment by a senior breast care doctor at the hospital where my original cancer was treated some 18 years ago. I feel that an earlier diagnosis may possibly have produced a kinder outcome.”
4	Wait for test/scan results	14	“Yes, I have a monthly appointment with my cancer doctor and the results of the CT’s are never in time.”
5	Appointment/operation delays and cancellations	13	“The major issue at [anon] concerned late cancellation of PET scans because the Gallium product was not available/failed quality when I was due for important scans.”

Unlike in the positive comments, the negative comments did not exhibit a ‘majority’ complaint (ie, a theme of feedback that occurred in over half of the comments analysed). However, over a third of patients (37%) complained about communication issues. The types of communication issues that were raised in the comments varied and included a mixture of complaints about poor communication from practitioners to patients (including how diagnoses of cancer were delivered; eg, being over the telephone) and breakdowns in communication between members of staff, departments and hospitals. In case of the latter, respondents frequently connected related issues around interdepartment and interhospital communication to perceived problems with the continuity and coordination of care across departments and providers.

Three of the themes of negative feedback relate to patient concerns around long waits (long waiting times for appointments and operations, long waits for test and scan results, and delays and cancellations to appointments and operations). Cumulatively, these complaints occurred across almost half (49%) of the comments in the sample. Such comments could be interpreted as an inverse of the types of positive comments about speed and efficiency noted previously.

The final theme of negative feedback, treatment, was observed in 18% of comments. Of the sample of positive comments analysed previously, 29% identified standards of treatment as an area for praise. Patients tended to complain about treatment standards when they perceived that practitioners (usually general practitioners) failed to diagnose cancer during an initial appointment.

## Discussion

On a scale of 0–10, the patients in this sample were most likely to give the most positive score of 10, while the overwhelming majority gave a score of at least 8 out of 10. Overall, then, this indicates that the vast majority of patients who provided comments are reasonably satisfied with the cancer care they received and gave generally positive feedback. Our qualitative analysis of 1000 positive comments found that the majority of respondents praised staff’s interpersonal skills. This strong focus on staff members’ interpersonal skills in positive feedback about cancer services is consistent with previous studies which have found that this theme tends more than others to drive more general healthcare service feedback.^16 17^ Other frequent themes in positive feedback included treatment standards, staff’s communication skills, speed of diagnosis and treatment, and staff members’ technical competence. The most prominent themes in negative comments were communication skills, treatment standards and waiting times for appointments and test/scan results, and delays and cancellations to appointments and operations.

When we compare the top 5 themes of positive and negative evaluations, we can observe some notable similarities and differences. Standards of treatment emerged as frequent themes in both positive and negative feedback, although it was more likely to be the target of praise than criticism, and this theme could also be linked to staff’s technical competence, which also featured among the most frequent themes of the positive comments. Communication skills also constituted a frequent theme of both the positive and negative evaluations. Aspects relating to communication are thus highly valued by patients accessing cancer care services, driving both praise and criticism. While this theme made up a larger portion of the negative than positive feedback, we should also bear in mind that the positive comments are, in raw terms, more frequent than the negative ones. So, while communication skills make up a larger proportion of the negative feedback, this does not necessarily mean that they are more likely to be evaluated negatively than positively overall.

A striking difference between the themes of the positive and negative feedback is the comparative lack of focus on staff’s interpersonal skills in the latter relative to the former. We could interpret this as an extremely positive endorsement for the interpersonal skills of the staff involved in the delivery of NHS cancer care services in England, particularly when we consider that previous research on general patient feedback has found staff’s interpersonal skills to be a prominent driver not only of positive but also of negative feedback.^16 17^

A substantial proportion of respondents’ evaluations focused on issues around time and waiting. This theme was more characteristic of negative feedback, where approximately half of the negative comments analysed complained about long waits for appointments and operations, and for results of scans and tests, and delays and cancellations to appointments, which then resulted in long waits. The theme of waits has, as noted, been observed in previous research on patient feedback on cancer services. Lengthy waits are likely an outcome of a mismatch between demand and resources, where funding levels of UK healthcare services have, in recent years, failed to keep up with increasing demand on those services.^15^ Addressing such concerns is therefore not straightforward and is a challenge that requires a response from policymakers and healthcare system organisers. However, for those more directly responsible for ‘front line’ cancer care service delivery, the complaints indicated some practical steps that could be taken in the ways that such lengthy waits are handled and communicated. For example, experiences of long waits seemed to be exacerbated in cases where patients were not informed about the length of waits and delays and cancellations in a timely manner. Therefore, communicating cancellations, delays and anticipated long waits to patients early and in a clear way may help to set more realistic expectations.

A strength of the mixed methods analytical approach taken in this study is that it has usefully allowed us to not only identify broad quantitative patterns in the ratings given by respondents, but through closer qualitative analysis to also understand the themes which drive those evaluations. This has been of value in terms of identifying areas of patient priority, but also in terms of understanding where patients perceive providers to be performing well and areas where they feel there could be improvement and what those improvements should be (discussed earlier in this section). While helpful in this regard, a limitation of this more qualitative analysis is that it is necessarily less scalable than the quantitative analysis which preceded it, which was able to account for generalisable trends in a way that our sample-based qualitative analysis could not.

While this study was based on the largest and most recent collection of feedback on NHS cancer care services in England, a limitation of this data set pertains to demographic imbalance, as some groups are under-represented in the Cancer Patient Experience Survey relative to others. For example, people from lesbian, gay, bisexual, transgender, queer (or sometimes questioning) and other (LGBTQ+) backgrounds make up 1% of the comments, while people who speak English as an additional language constitute 3.5% of the feedback analysed. Another imbalance in the data relates to respondents’ ethnicity, with 87.09% of responses representing the perspectives of respondents identifying as white English/Welsh/Scottish/Northern Irish. The NHS is establishing a working group on this matter. Future research should aim to identify ways in which such imbalances might be redressed, such as analysing minority group data separately with more detailed qualitative analyses or comparing equal-sized samples.

## Data Availability

No data are available.
